# Conventional Therapies Do Not Prolong the Prognosis of Hepatocellular Carcinoma Patients with Extrahepatic Metastases under Receiving of Tyrosine Kinase Inhibitors

**DOI:** 10.3390/cancers14030752

**Published:** 2022-01-31

**Authors:** Hiroshi Maeda, Kouichi Miura, Naoki Morimoto, Shunji Watanabe, Mamiko Tsukui, Yoshinari Takaoka, Hiroaki Nomoto, Rie Goka, Naoto Sato, Kazue Morishima, Yasunaru Sakuma, Naohiro Sata, Noriyoshi Fukushima, Norio Isoda, Hironori Yamamoto

**Affiliations:** 1 Department of Medicine, Division of Gastroenterology, Jichi Medical University, 3311-1 Yakushiji Shimotsuke, Tochigi 329-0498, Japan; r1039hm@jichi.ac.jp (H.M.); morimoto@jichi.ac.jp (N.M.); 95103sw@jichi.ac.jp (S.W.); tsukui@jichi.ac.jp (M.T.); r0830yt@jichi.ac.jp (Y.T.); r0843hn@jichi.ac.jp (H.N.); m04027rg@jichi.ac.jp (R.G.); r1320sn@jichi.ac.jp (N.S.); isodano1@jichi.ac.jp (N.I.); ireef@jichi.ac.jp (H.Y.); 2 Department of Surgery, Division of Gastrointestinal, General and Transplant Surgery, Jichi Medical University, 3311-1 Yakushiji Shimotsuke, Tochigi 329-0498, Japan; morishim@jichi.ac.jp (K.M.); naruchan@jichi.ac.jp (Y.S.); sata2018@jichi.ac.jp (N.S.); 3 Department of Pathology, Jichi Medical University, 3311-1 Yakushiji Shimotsuke, Tochigi 329-0498, Japan; nfukushima@jichi.ac.jp

**Keywords:** hepatocellular carcinoma, extrahepatic metastases, prognosis, tyrosine kinase inhibitor, conventional therapy

## Abstract

**Simple Summary:**

Tyrosine kinase inhibitors (TKIs), including sorafenib and lenvatinib, have been the current standard treatment for advanced hepatocellular carcinoma (HCC) in cases where an immune checkpoint inhibitor cannot be used. The SHARP study showed that sorafenib tended to be less effective for extrahepatic metastases than for vascular invasion. Moreover, lenvatinib showed a response similar to that of sorafenib in such patients. The aforementioned data suggested that the addition of conventional therapies, including chemoembolization and radiation therapy, may improve the prognosis of such patients. Our retrospective study found that TKI promoted a longer overall survival in patients with extrahepatic metastases compared to conventional therapies. TKI plus conventional therapies did not promote a better prognosis compared to TKI alone. Thus, conventional therapies can be an option when events that worsen the quality of life occur in HCC patients with extrahepatic metastases.

**Abstract:**

Background: Conventional therapies, including chemoembolization and radiation therapy, have been expected to prolong the prognosis of hepatocellular carcinoma (HCC) patients with extrahepatic metastases, which remains poor. However, little information is available on the efficacy of conventional therapies for such patients under tyrosine kinase inhibitor (TKI) treatment. Methods: We retrospectively investigated 127 HCC patients with extrahepatic metastases, who were divided into the non-TKI (conventional therapies) and TKI groups and further subdivided into the TKI alone and TKI plus conventional therapies groups. Conventional therapies included transcatheter arterial chemoembolization, cisplatin-based chemotherapy, radiation, surgery, and UFT, an oral chemotherapeutic agent. Results: The median of the overall survival (OS) of the 127 patients with extrahepatic metastases was 7.0 months. Meanwhile, the median OS of the TKI and non-TKI groups was 12.1 and 4.1 months, respectively. Imitating TKI after diagnosing metastases promoted a favorable increase in OS. Among the TKI group, the median OS in the TKI alone group was 8.9 months. TKI plus conventional therapies promoted no improvement in OS after adjusting for the patients’ background data. Conclusion: TKI promoted a better OS in HCC patients with extrahepatic metastases compared to conventional therapies. However, TKI plus conventional therapies promoted no improvement in the prognosis of such patients.

## 1. Introduction

Hepatocellular carcinoma (HCC) is the sixth most common cancer and the third leading cause of cancer death worldwide. In 2020, 905,677 new cases and 830,180 deaths had been reported according to a global cancer report [1]. Thus, the World Health Organization has sought to reduce the number of patients with HCC. Given that HCC is characterized by multi-focal cancer, it often recurs even after curative treatments, including surgical resection and radiofrequency ablation. Finally, HCC can progress to advanced stages, featuring major vessel invasion and/or extrahepatic metastases. Notably, the prognosis of patients whose HCC has progressed to the advanced stages is extremely poor.

Current standard treatments for advanced HCC include tyrosine kinase inhibitors (TKIs) and a combination of atezolizumab and bevacizumab, an immune checkpoint inhibitor (ICI) and a monoclonal antibody against vascular endothelial growth factor (VEGF), respectively. Before TKIs and ICI were available, patients with extrahepatic metastases were treated with conventional methods, including resection, radiation, and chemotherapies through the hepatic artery. Considering that intrahepatic lesions are a key prognostic factor, transcatheter arterial chemoembolization (TACE) and/or cisplatin-based chemotherapy had often been used. Indeed, reports have shown that cisplatin-based chemotherapy prolonged the prognosis of patients with advanced HCC [2,3]. When the primary lesions are well controlled, surgical resection can be performed for extrahepatic metastases, including the lungs, lymph nodes, and adrenal glands [4,5,6]. Radiation therapy for bone metastases has been reported to improve the prognosis [7]. However, the median survival time of patients with extrahepatic metastases has been reported to be 4.9–7.0 months, with a 1 year survival rate of 21.7–24.9% [8,9].

The first large study on sorafenib for patients with advanced HCC (the SHARP study) showed a median survival time of 10.7 months, which was significantly longer than that of the placebo [10]. Meanwhile, the first large study on lenvatinib (the REFLECT study) found a median survival time of 13.6 months, which was not inferior to that of sorafenib [11]. Thus, either sorafenib or lenvatinib has been used at the first-line TKI for advanced HCC. Although sorafenib started as the standard treatment for patients with advanced HCC, the SHARP study found that sorafenib tended to be less effective against extrahepatic metastases than against vascular invasion [10]. This trend was also noted in an analysis on Asian-Pacific patients [12]. Moreover, lenvatinib had a response that was similar to that of sorafenib in the treatment for HCC involving portal vein and/or extrahepatic spread. Although patients with extrahepatic metastases seemed to have longer OS after sorafenib was available [13], detailed information focusing on extrahepatic metastases was lacking. Furthermore, the outcomes of patients who received conventional therapies under TKI treatments remain unknown.

The present study therefore aimed to compare (1) TKI versus conventional therapies, (2) TKIs alone versus TKI plus conventional treatments, and (3) sorafenib versus lenvatinib, as well as determine (4) the efficacy of TKI against extrahepatic lesions in HCC patients with extrahepatic metastases. To this end, we retrospectively investigated the OS of HCC patients with extrahepatic metastases who received TKIs and conventional therapies.

## 2. Materials and Methods

### 2.1. Patients and Study Design

We retrospectively investigated 825 patients with HCC who visited Jichi Medical University Hospital from 1 May 2009 to 31 December 2019. During the study period, 127 cases with extrahepatic metastases were identified. No exclusion criteria were established. The overall survival (OS) and 1 and 3 year survival rates were assessed from the date at which extrahepatic metastases were detected until death from any cause. Performance status was determined according to Eastern Cooperative Oncology Group score. This study was approved the by ethics committee of Jichi Medical University (A19-154). All procedures followed have been performed in accordance with the ethical standards stated in the 1964 Declaration of Helsinki and its later amendments.

### 2.2. Diagnosis of HCC and Extrahepatic Metastases

HCC was diagnosed by hepatologists and radiologists using contrast-enhanced computed tomography and/or magnetic resonance imaging. Histologically proven HCC were also included. Moreover, positron emission tomography, bone scintigraphy, and X-ray examinations were used to diagnose extrahepatic metastases. Vascular invasion was defined as invasion of the portal vein (≥Vp1), hepatic vein (≥Vv1), and inferior vena cava on CT examinations.

### 2.3. Treatment and Responses

In the present study, 67 patients received TKI, including sorafenib and lenvatinib, as their first-line treatment. Conventional therapies included TACE, cisplatin-based chemotherapy using transcatheter arterial infusion (TAI), radiation, resection, and oral UFT (a combination of tegafur and uracil). In TACE, epirubicin hydrochloride, miriplatin hydrate, and cisplatin were used with ethyl ester of iodinated poppy-seed oil fatty acid (lipiodol^®^). Treatment decisions were made by each investigator. Patients who received TKI continued the therapy until the occurrence of grade 2 or higher adverse events (CTGAE, version 5.0 criteria), progressive disease (PD) according to Response Evaluation Criteria in Solid Tumors (RECIST), or death. Treatment response was evaluated as complete response (CR), partial response (PR), stable disease (SD), and PD based on RECIST version 1.1 [14]. Disease control rate (DCR) was a sum of CR, PR, and SD. Although imaging examinations were not always performed routinely, the first imaging test after the diagnosis of extrahepatic lesions was used for the assessment of efficacy. For the assessment of extrahepatic metastases, the maximum size of tumor was evaluated according to RECIST version 1.1.

### 2.4. Statistical Analysis

Patient background data were analyzed using the chi-square test, *t*-test, and Mann–Whitney test. Cumulative survival rates were assessed using the Kaplan–Meier survival curve, and differences were evaluated using the log rank test. Statistical analyses were performed using Stata/IC 15.1 (STATA Corporation, College Station, TX, USA), with *p* values < 0.05 indicating statistical significance.

## 3. Results

### 3.1. Characteristics of Patients with HCC Diagnosed with Metastases

Among the 825 patients with HCC, 127 (15.4%) were diagnosed with extrahepatic metastases (Table 1). The median age of the patients with extrahepatic metastases, 80.3% of whom were men, was 69.4 years old. The most common etiology was HCV, followed by HBV and alcohol. No significant differences in etiologies were observed between patients with and without metastases (data not shown). During the study period, a total of 167 extrahepatic metastatic sites were noted, with the most frequent organ involved being the bones (56 sites, 33.5%), followed by the lymph nodes (50 sites, 30.0%), lungs (47 sites, 28.1%), and adrenal grands (7 sites, 4.2%).

### 3.2. Prognosis of Patients with Extrahepatic Metastases

**Figure 1:**
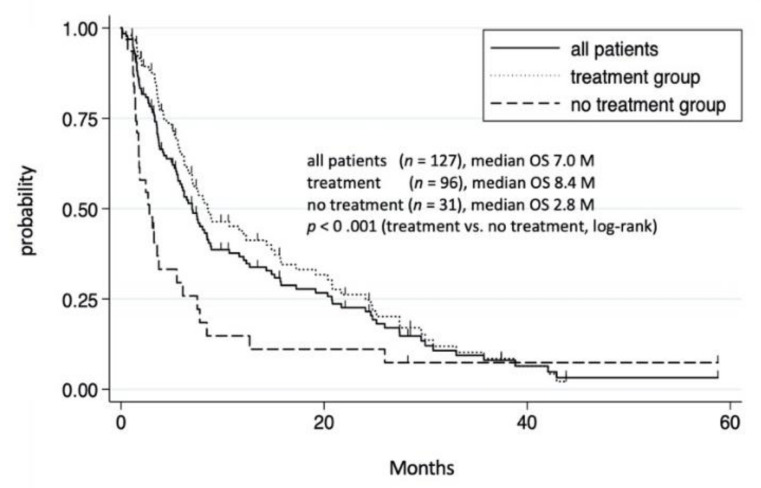
Cumulative survival rate of 127 HCC patients with extrahepatic metastases. Survival rates of all patients with extrahepatic metastases at 1 and 3 years were 36.4% and 7.9%, respectively. In treatment group, survival rates at 1 and 3 years were 43.5% and 8.6%, respectively. In the non-treatment group, survival rates at 1 and 3 years were 15.5% and 7.8%, respectively.

We then investigated the OS of the 127 patients with extrahepatic metastases. During the study period, 103 patients died, 19 survived, and 5 were unknown. The median OS of all patients with extrahepatic metastases was 7.0 months, with 1 and 3 year survival rates of 36.4% and 7.9%, respectively (Figure 1).

Among the 127 patients with extrahepatic metastases, 96 received treatments, including TKI and/or conventional therapies, whereas the remining 31 received no treatment. Those who received no treatment had a significantly shorter OS than those who did (*p* < 0.001). The median OS in the treatment and no treatment groups were 8.4 and 2.8 months, respectively, partially due to the unfavorable conditions in the no treatment group, including a poor liver function reserve and an advanced HCC stage (Table S1).

### 3.3. Prognosis of Patients Who Received TKIs

**Figure 2:**
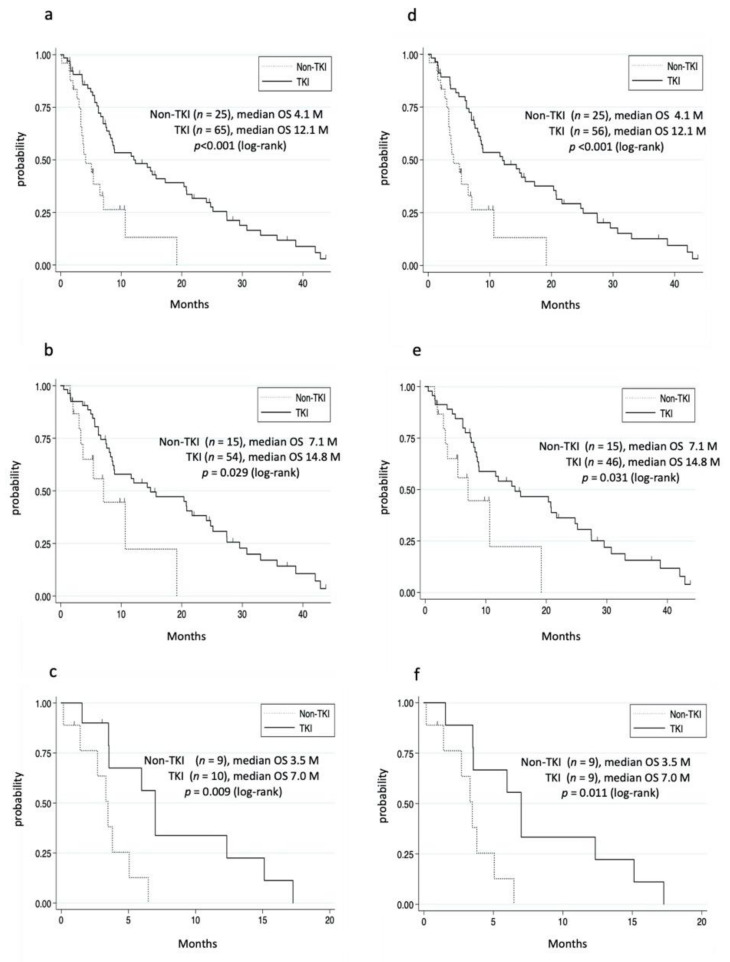
Cumulative survival rate of the non-TKI and TKI groups. (**a**–**c**) Patients with extrahepatic metastases who used TKI. (**a**) In the TKI group, survival rates at 1 and 3 years were 51.5% and 11.7%, respectively. In the non-TKI group, survival rates at 1 and 3 years were 13.1% and 0%, respectively. (**b**) Child–Pugh class A. In the TKI group, the survival rates at 1 and 3 years were 55.9% and 14.2%, respectively. In the non-TKI group, the survival rates at 1 and 3 years were 22.4% and 0%, respectively. (**c**) Child–Pugh class B. In the TKI group, the survival rates at 1 and 3 years were 33.8% and 0%, respectively. In the non-TKI group, the 1 year survival rate was 0%. (**d**–**f**) Patients who started TKI after being diagnosed with extrahepatic metastases. (**d**) The survival rate of the TKI group at 1 and 3 years were 51.4% and 12.6%, respectively. (**e**) Child–Pugh class A. The survival rates of the TKI group at 1 and 3 years were 56.5% and 15.7%, respectively. (**f**) Child–Pugh class B. The survival rates of the TKI group at 1 and 3 years were 33.3% and 0%, respectively.

We then investigated the effects of treatments in patients with extrahepatic metastases. Accordingly, the 96 patients who received treatments were divided into the TKI group (TKI alone or TKI plus conventional therapies) and non-TKI group (conventional therapies). Two patients who received pembrolizumab or ramucirumab were excluded from the TKI group. Moreover, four patients who received TKIs before the occurrence of metastases were excluded from non-TKI group. Thus, we compared the OS between 65 patients in the TKI group and 25 patients in the non-TKI group. Details regarding the TKI and conventional therapies are shown in Table S2. The median OS in the TKI and non-TKI groups were 12.1 and 4.1 months, respectively, the difference being statistically significant (Figure 2a). Although the TKI group had a significantly superior prognosis compared to the non-TKI group, patients in the TKI group had a more favorable condition, including a liver function reserve (Table S3). To adjust for the patient’s background data, we analyzed the OS among patients with Child–Pugh classes A and B. The TKI group had a significantly longer OS than the non-TKI group, even after adjusting for the patient background (Figure 2b,c, Table 2). Among the 65 patients in the TKI group, 56 started TKI after the diagnosis of extrahepatic metastases. Patients who started TKI after having been diagnosed with extrahepatic metastases had a significantly longer median OS than those in the non-TKI group (Figure 2d). Moreover, the TKI group had a significantly superior OS compared to the non-TKI group among patients with Child–Pugh classes A and B (Figure 2e,f, Tables S4 and S5).

### 3.4. Prognosis of Patients Who Received TKI plus Conventional Therapies

**Figure 3:**
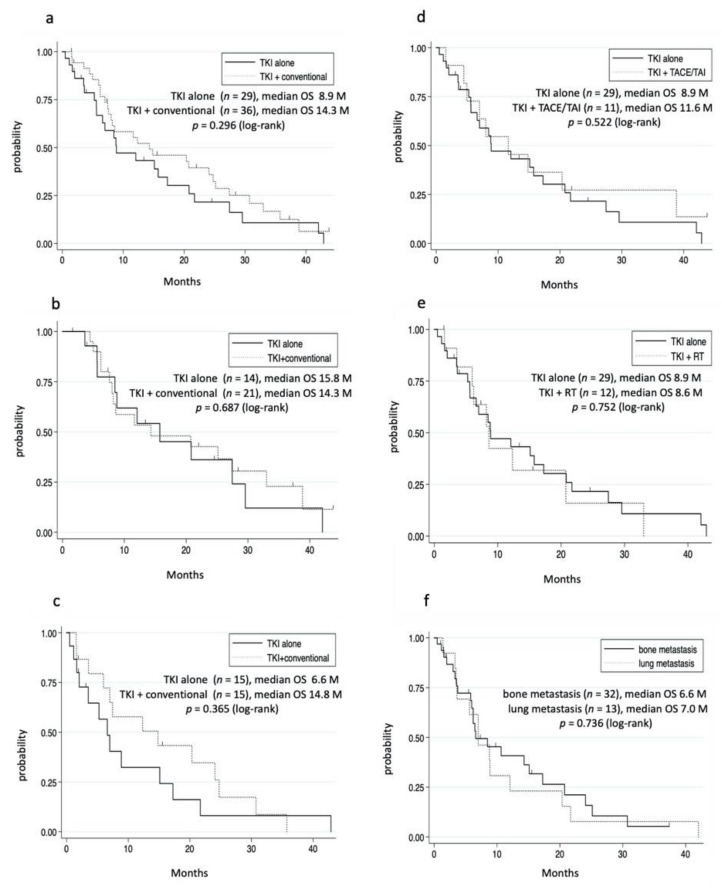
Cumulative survival rate. (**a**–**c**) TKI alone versus TKI plus conventional therapies. (**a**) All patients in TKI group. In the TKI alone group, survival rates at 1 and 3 years were 46.7% and 10.7%, respectively. In the TKI plus conventional therapies group, survival rates at 1 and 3 years were 55.0% and 12.6%, respectively. (**b**) REFLECT IN. In the TKI alone group, survival rates at 1 and 3 years were 61.8% and 12.1%, respectively. In the TKI plus conventional therapies group, survival rates at 1 and 3 years were 53.2% and 22.7%, respectively. (**c**) REFLECT OUT. In the TKI alone group, survival rates at 1 and 3 years were 32.4% and 8.1%, respectively. In the TKI plus conventional therapies group, survival rates at 1 and 3 years were 57.6% and 0%, respectively. (**d**) TKI alone versus TKI plus TACE/TAI. In the TKI plus TACE/TAI group, survival rates at 1 and 3 years were 45.3% and 27.3%, respectively. (**e**) TKI alone versus TKI plus radiation. In the TKI plus irradiation group, survival rates at 1 and 3 years were 42.4% and 0%, respectively. (**f**) Lung versus bone metastases. In the lung metastases group, survival rates at 1 and 3 years were 30.6% and 7.5%, respectively. In the bone metastases group, survival rates at 1 and 3 years were 40.7% and 5.1%, respectively.

Among the 65 patients in the TKI group, 36 received TKI plus conventional therapies. Although the TKI plus conventional therapies group tended to have a long OS, the difference was not significant (Figure 3a, Table 3). Then, we compared the OS in patients who met the REFLECT criteria (REFLECT IN) and did not meet the REFLECT criteria (REFLECT OUT) [11]. The major exclusion criteria were HCC occupation ≥ 50% of the liver, obvious invasion of the bile duct, or invasion at the main portal vein (Vp4). In the REFLECT IN, TKI plus conventional therapies had almost the same OS as the TKI alone group (Figure 3b). In the REFLECT OUT, although the TKI plus conventional therapies group tended to have a longer OS, the backgrounds of the patients were favorable (Figure 3c, Table S6). Considering that conventional therapies include several therapies, we separately evaluated TACE/TAI and radiation therapy. The TKI plus TACE/TAI group had almost the same OS as the TKI alone group (Figure 3d). Additionally, the TKI plus radiation group had the same OS as the TKI alone group (Figure 3e). Furthermore, TKI plus multiple conventional therapies did not prolong the OS even after adjusting for the patients’ background (data not shown). Oral UFT is a systemic chemotherapy, which may contribute to the OS in the TKI plus conventional therapies group. When patients who used oral UFT were excluded, the OS was similar between the TKI alone group and TKI plus conventional therapies group (data not shown). The majority of the patients in the TKI plus TACE/TAI group and TKI plus radiation group had lung metastases and bone metastases, respectively. No significant differences in prognosis were observed between those with lung metastases and bone metastases (Figure 3f and Table S7).

### 3.5. Effect of Sorafenib and Lenvatinib in Patients with Extrahepatic Metastases

**Figure 4:**
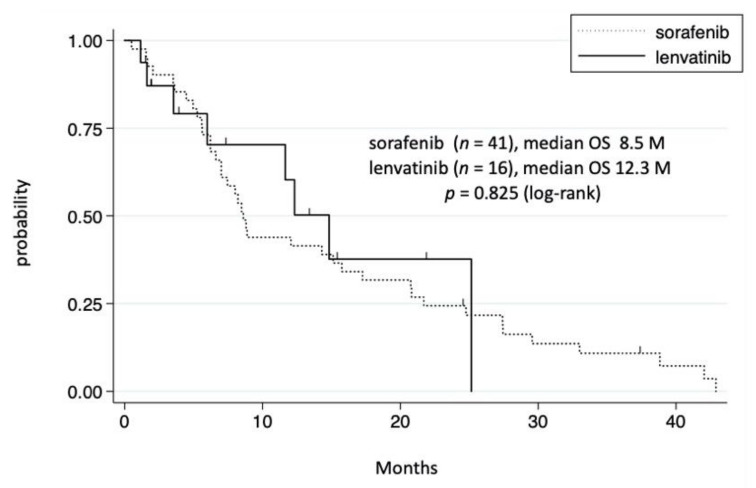
Cumulative survival rate of the sorafenib and lenvatinib groups. In the sorafenib group, survival rates at 1 and 3 years were 44.0% and 11.0%, respectively. In the lenvatinib group, survival rates at 1 and 3 years were 60.3% and 0%, respectively.

Among the 65 patients in the TKI group, 41 and 16 received sorafenib and lenvatinib, respectively, whereas the remaining 8 received two or three TKIs (Table 4). We initially compared the prognosis between the sorafenib and lenvatinib groups, both of which included TKI alone and TKI plus conventional therapies. No significant difference in prognosis was noted between the sorafenib and lenvatinib groups (Figure 4). Moreover, no significant difference in OS was observed between the sorafenib plus conventional therapies group (*n* = 17) and lenvatinib plus conventional therapies group (*n* = 12) (data not shown). The median treatment duration in patients receiving TKI, sorafenib, and lenvatinib was 4.1, 4.4, and 1.4 months, respectively. Adverse events and PD tended to be the most frequent cause for discontinuing lenvatinib and sorafenib treatment, respectively (Table 4). We also investigated the difference in OS between single and multiple TKIs. Although patients who received multiple TKIs tended to have a longer OS, no significant difference was observed after adjusting for the patient’s background (data not shown).

### 3.6. Effect of TKIs on Extrahepatic Metastases

**Figure 5:**
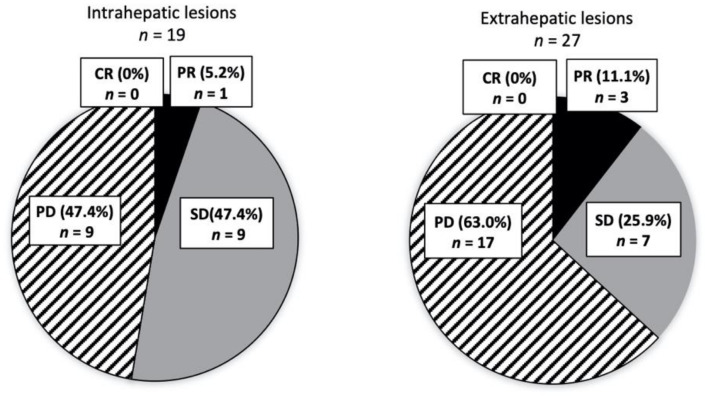
Response to TKI. The efficacies of TKI for intrahepatic (*n* = 19) and extrahepatic lesions (*n* = 27) were separately evaluated. Numbers of each extrahepatic lesion are as follows; PR, LN (lymph node) 2, Other 1; SD, Bone 3, Lung 2, LN 1, Other 1; PD, Bone 2, Lung 8, LN 3, Other 4.

Among the 29 patients in the TKI alone group, 19 had data on the DCR of intrahepatic and extrahepatic lesions. We analyzed 19 intrahepatic lesions and 27 extrahepatic lesions in patients who used TKIs after the diagnosis of extrahepatic metastases. The DCRs of intrahepatic and extrahepatic lesions were 52.6% and 37.0%, respectively (Figure 5). No significant difference in efficacy was observed between intrahepatic and extrahepatic lesions (*p* = 0.328).

## 4. Discussion

The present study demonstrated that TKI treatment provided a longer OS compared to conventional therapies. However, TKI plus conventional therapies did not prolong the prognosis compared to TKI alone. No significant difference in OS was observed between patients receiving sorafenib and lenvatinib. TKIs were as effective against extrahepatic metastases as with intrahepatic lesions.

The prognosis of patients with extrahepatic metastases still remained poor when TKI could be administered. The present study found that the median OS of patients with extrahepatic metastases was 7.0 months, which was almost equal to that in the pre-TKI era [8,15,16]. This is because 24% of patients did not receive any treatments for HCC given the poor hepatic function reserve and/or performance status at the time of extrahepatic metastases. Additionally, 20% of the patients received conventional therapies used in the pre-TKI era. Indeed, the median OS of patients in the no treatment group and non-TKI group were 2.8 and 4.1 months, respectively. Thus, nearly half of the patients in the present study could be characterized as being in a situation similar to that of the pre-TKI era. Our group had often used conventional therapies according to the patient’s requests just after sorafenib was available. However, our data showed that, among HCC patients with extrahepatic metastases, the non-TKI group (conventional therapies group) had a significantly inferior prognosis compared to the TKI group.

In contrast to the pre-TKI era, the present study showed higher 1 and 3 year survival rates in patients with extrahepatic metastases compared to previous reports [8,15]. We believe that these survival rates were caused by the TKI group, which had 1and 3 year survival rates of 51.5% and 11.7%, respectively. Moreover, our data showed that the TKI group had a median OS of 12.1 months, which agreed with the previous reports after sorafenib was available wherein the median OS was 10.3–11 months [13,17]. In a comparison of the OS, we had to pay attention to eligible criteria because they varied among studies. When we adjusted the eligible criteria to the REFLECT study [11], the median OS of patients with extrahepatic metastases in our study was 15.8 months, which was longer than that of the REFLECT study (sorafenib 9.8 months, lenvatinib 11.5 months). However, the number of patients in our study was small compared to that of the REFLECT study (*n* = 14 versus *n*= 329–336). TKI alone provided a similar DCR of intrahepatic and extrahepatic lesions. The TACTICS trial [18] revealed that sorafenib plus TACE promoted a significantly better progression-free survival of patients with unresectable HCC compared to TACE alone. Although the TACTICS trial did not include patients with extrahepatic metastases, sorafenib prolonged the development of major vascular invasion and extrahepatic metastases. These data suggested that TKIs can prolong the prognosis of HCC by delaying the progression of HCC, including extrahepatic metastases.

Our data showed that the combination of TKI and conventional therapies provided no significant benefit in prognosis compared to TKI alone. It is of interests that conventional therapies contributed to the prognosis under TKI treatment. In the SHARP [10] and the REFLECT studies [11], patients with favorable conditions, such as Child–Pugh class A, were enrolled in the clinical trials. However, we frequently encounter patients who do not meet the eligible criteria of such studies due to a “far advanced stage” in clinical practice. Although TKI plus conventional therapies tended to have a longer OS than TKI alone in the “far advanced stage”, here defined as REFLECT OUT, our data failed to show a prolonged prognosis. Among conventional therapies, TACE/TAI has been used to control intrahepatic HCC, even in patients with extrahepatic metastases, given that the intrahepatic tumor stage has been identified as a prognostic factor [8]. Previous retrospective studies have reported that cisplatin-based chemotherapy via a catheter can prolong the prognosis of patients with advanced HCC [2,3]. In contrast, a prospective study revealed that sorafenib plus cisplatin-based chemotherapy failed to improve the prognosis of patients with advanced HCC compared to those treated with sorafenib alone [19]. Considering that extrahepatic metastatic lesions were noted at multiple sites, we investigated the OS provided by each treatment, namely TKI plus TACE/TAI, TKI plus radiation therapy, and TKI plus multiple conventional therapies. These data suggested that conventional therapies did not provide sufficient prognostic benefits among patients with extrahepatic metastases, particularly with regard to controlling intrahepatic and extrahepatic lesions.

Our study included patients treated with lenvatinib, which may have contributed to the favorable prognosis of patients with extrahepatic metastases. However, there was no significant difference in the OS between sorafenib and lenvatinib groups. Although lenvatinib has been reported to promote a longer progression-free survival compared to sorafenib, the OS between sorafenib and lenvatinib in patients with advanced HCC was almost equal [20,21]. Currently, limited data are available on the comparison of these TKIs in patients with extrahepatic metastases. Our data showed no superiority of lenvatinib over sorafenib for patients with extrahepatic metastases. However, the majority of the patients of the TKI group received sorafenib (75%, 49 patients). Moreover, the treatment duration of lenvatinib was short due to adverse events. Further investigations are required to clarify this matter.

In the present study, the most common site of extrahepatic metastases was the bones. According to previous reports, the most common site of extrahepatic metastases was the lungs [15,16,22]. This could have been partly attributed to the characteristics of our hospital, in which, patients with bone metastases are referred for radiation therapy. Although some reports demonstrated that bone metastases had a better prognosis compared to other metastatic sites, [23,24] mixed data have been reported regarding the prognosis among metastatic sites [25]. Notably, our data showed no significant difference between bone and lung metastases.

The present study has a few limitations worth noting. Given the retrospective nature of this study and our inclusion of extrahepatic lesions, conventional therapies included multiple modalities, single to multiple sessions, and combinations thereof. Indeed, some patients had huge intrahepatic lesions and multiple sites of extrahepatic metastases. In these cases, TACE was added to prevent the rupture of the HCC. In addition, radiation therapy for bone metastases was added to relieve pain. As such, it was difficult to compare the efficacy between a single therapy and TKI, which should have been ideal. The present study failed to show any favorable results for the use of conventional therapies. However, we do not always deny the use of conventional therapies, which can be one option in the event that the quality of life worsens.

The recently IMbrave150 study demonstrated that the effects of an ICI coupled with a VEGF inhibitor were superior to that of sorafenib in patients with advanced HCC [26]. In patients with extrahepatic metastases, the atezolizumab plus bevacizumab had a longer survival than sorafenib (median OS, 17.8 versus 10.4 month) [27]. Thus, treatment with an ICI coupled with a VEGF inhibitor has recently become the standard treatment for patients with extrahepatic metastases. Although the eligible criteria between the IMbrave150 study and our study are different, the median OS of our subgroups (14.8 months in Child–Pugh class A, Figure 2b, 15.8 months in REFLECT IN, Figure 3b) was close to that of the IMbrave150 study. In a retrospective study, a triple combination of an ICI, TKI, and TACE showed favorable outcomes compared with a dual combination of an ICI and TKI [28]. Currently, multiple clinical trials are taking place using ICI-based therapies, which include a conventional therapy such as TACE, for unresectable HCC (https://clinicaltrials.gov/ct2/home, accessed on 23 December 2021). Our study may highlight the position of conventional therapies in the era of ICI therapy. Further studies are nonetheless necessary to determine the best treatment option for HCC patients with extrahepatic metastases.

## 5. Conclusions

TKI promoted a better prognosis in HCC patients with extrahepatic metastases compared to conventional therapies. Conventional therapies did not prolong the prognosis of HCC patients with extrahepatic metastases under the receiving of tyrosine kinase inhibitors.

## Figures and Tables

**Table 1:** Characteristics of patients with hepatocellular carcinoma upon diagnosis of metastases.

Number	127
Age (years old)	69.4 (25.4–87.0)
Men	102 (80.3%)
HBV/HCV/HBV + HCV Alcoholic/NASH/Others	19 (15.0%)/69 (54.3%)/3 (2.4%) 15 (11.8%)/12 (9.4%)/9 (7.1%)
FIB4–index	5.07 (0.48–39.5)
Child–Pugh score	6 (5–13)
ALBI score	−2.15 (−3.26 to −0.62)
AFP (ng/mL)	155 (1–671,940)
DCP (mAU/mL)	575 (10–1,084,600)
Frequency of multiple intrahepatic HCC	95 (74.8%)
Maximum size of intrahepatic HCC (mm)	44 (0–154)
Vascular invasion	80 (63.0%)
Hepatic TNM	4 (0–4)
Performance status	0 (0–4)
Number of metastatic sites Bone/Lymph node/Lung/Adrenal glands/Peritoneum/Others	167 56 (33.5%)/50 (30.0%)/47 (28.1%) 7 (4.2%)/4 (2.4%)/3 (1.8%)

AFP, α-fetoprotein; DCP, des-γ-carboxy prothrombin; HBV, hepatitis B virus; HCV, hepatitis C virus; NASH, nonalcoholic steatohepatitis; TNM, tumor–node–metastasis.

**Table 2:** Background data of patients in the TKI and non-TKI groups.

Features	Child–Pugh A			Child–Pugh B		
	TKI	Non-TKI	*p*-Value	TKI	Non-TKI	*p*-Value
Number	54	15		10	9	
Age (years old)	69.6 (25.4–85.9)	75.5 (61.8–83.4)	0.163	72.5 (51.9–83.4)	63.7 (49.6–87.0)	0.270
Men	44 (81.5%)	11 (73.3%)	0.548	9 (90.0%)	7 (77.8%)	0.466
FIB-4 index	3.94 (0.48–27.8)	4.43 (1.74–14.3)	0.490	6.42 (3.75–17.2)	12.3 (3.58–25.7)	0.125
ALBI score	−2.49 (−3.25 to −1.50)	−2.38 (−3.26 to −1.65)	0.258	−1.87 (−2.21 to −1.19)	−1.77 (−2.44 to −0.90)	0.606
AFP (ng/mL)	52 (1–128,650)	155 (4–369,127)	0.230	796 (2–123,578)	577 (2–671,940)	0.838
DCP (mAU/mL)	246 (10–1,084,600)	166 (16–562,056)	0.436	1066 (23–82,003)	5952 (24–45,363)	0.744
Frequency of multiple intrahepatic HCC	37 (68.5%)	10 (66.7%)	0.918	7 (70.0%)	9 (100%)	0.073
Maximum size of intrahepatic HCC (mm)	27 (0–154)	41 (0–125)	0.301	52 (0–127)	53 (24–138)	0.803
Vascular invasion	22 (40.7%)	10 (66.7%)	0.088	7 (70.0%)	8 (88.9%)	0.313
Hepatic TNM	3 (0–4)	3 (0–4)	0.162	3 (0–4)	4 (3–4)	0.030
Performance status	0 (0–3)	0 (0–4)	0.675	0 (0–2)	0 (0–3)	0.206

AFP, α-fetoprotein; DCP, des-γ-carboxy prothrombin; TKI, tyrosine kinase inhibitor; TNM, tumor–node–metastasis.

**Table 3:** Background data of patients who received TKI alone and TKI plus convention therapies.

Features	TKI Alone	TKI Plus Conventional Therapies	*p*-Value
Number	29	36	
Age (years old)	71.1 (25.9–85.9)	69.1 (49.9–85.1)	0.843
Men	25 (86.2%)	29 (80.6%)	0.546
FIB-4 index	5.88 (0.95–18.0)	3.93 (0.48–27.8)	0.240
Child–Pugh score	6 (5–11)	5 (5–8)	0.293
ALBI score	−2.32 (−3.20 to −0.90)	−2.43 (−3.25 to −1.19)	0.165
AFP (ng/mL)	169 (1–128,650)	21 (11–23,578)	0.083
DCP (mAU/mL)	395 (10–1,084,600)	394 (15–63,989)	0.932
Frequency of multiple intrahepatic HCC	21 (72.4%)	24 (66.7%)	0.618
Maximum size of intrahepatic HCC (mm)	47 (0–154)	24 (0–120)	0.030
Vascular invasion	17 (58.6%)	13 (36.1%)	0.070
Hepatic TNM	3 (0–4)	2 (0–4)	0.046
Performance status	0 (0–3)	0 (0–2)	0.260

AFP, α-fetoprotein; DCP, des-γ-carboxy prothrombin; TKI, tyrosine kinase inhibitor; TNM, tumor–node–metastasis.

**Table 4:** Background data of patients who received sorafenib or lenvatinib.

Features	Sorafenib Group	Lenvatinib Group	*p*-Value
Number	41	16	
Starting dose (mg) Sorafenib 200/400/600/800 Lenvatinib 4/8/12	8/21/2/10	6/4/6	
Discontinuation dose Sorafenib 200/400/600/800 Lenvatinib 4/8/12	9/16/8/8	8/5/3	
Age (years old)	71.1 (25.4–85.9)	67.8 (56.3–85.1)	0.638
Men	35 (85.4%)	12 (75.0%)	0.355
HBV	5 (12.2%)	3 (18.8%)	0.522
HCV	22 (53.7%)	11 (68.8%)	0.300
HBV + HCV	2 (4.9%)	0 (0.0%)	0.368
Alcoholic	6 (14.6%)	0 (0.0%)	0.106
NASH	2 (4.9%)	1 (6.2%)	0.835
Others	4 (9.7%)	1 (6.2%)	0.674
FIB-4 index	5.09 (1.56−18.0)	3.63 (0.48−14.7)	0.192
Child–Pugh score	5.5 (5–11)	5 (5 –7)	0.341
ALBI score	−2.42 (−3.25–0.90)	−2.43(−2.96–1.19)	0.986
AFP (ng/mL)	102 (1–128,650)	62 (1–123,578)	0.379
DCP (mAU/mL)	488 (10–1,084,600)	213 (18–63,989)	0.965
Frequency of multiple intrahepatic HCC	30 (73.2%)	10(62.5%)	0.429
Maximum size of intrahepatic HCC (mm)	45 (0–154)	35 (0–137)	0.449
Vascular invasion	21 (51.2%)	6 (37.5%)	0.351
Hepatic TNM	3 (0–4)	3 (0–4)	0.394
Performance status	0 (0–3)	0 (0–2)	0.334
TKI period	4.4 (0.2–40.9)	1.4 (0.2–26.9)	0.061
Reason of discontinuation PD/AE	14/24	2/14	0.074

AE, adverse event; AFP, α-fetoprotein; DCP, des-γ-carboxy prothrombin; HBV, hepatitis B virus; HCV, hepatitis C virus; NASH, nonalcoholic steatohepatitis; PD, progressive disease; TKI, tyrosine kinase inhibitor; TNM, tumor–node–metastasis.

## Data Availability

The data are presented within the paper. Additional raw data are available on request from the corresponding author.

## Supplementary Materials

The following supporting information can be downloaded at: https://www.mdpi.com/article/10.3390/cancers14030752/s1, Table S1: Background data of patients in the treatment and no treatment groups, Table S2: Contents of TKI and conventional therapies, Table S3: Background data of patients in TKI and non-TKI groups, Table S4: Background data of patients who started TKI after diagnosis of metastases, Table S5: Background data of Child-Pugh A and B patients who started TKI after diagnosis of metastases, Table S6: Background data of patients who meet the REFLECT criteria (REFLECT IN) and do not meet the criteria (REFLECT OUT), Table S7: Background data of patients with lung or bone metastases.

## Abbreviations

CR	complete response
HCC	hepatocellular carcinoma
ICI	immune checkpoint inhibitor
PD	progressive disease
PR	partial response
RECIST	Response Evaluation Criteria in Solid Tumors
SD	stable disease
TACE	transcatheter arterial chemoembolization
TAI	transhepatic arterial infusion
TKI	tyrosine kinase inhibitor
TNM	tumor–node–metastasis
VEGF	vascular endothelial growth factor
