# Supplementary material for: Conventional Therapies Do Not Prolong the Prognosis of Hepatocellular Carcinoma Patients with Extrahepatic Metastases under Receiving of Tyrosine Kinase Inhibitors
Source: Cancers (Basel). 2022 Jan 31;14(3):752. doi: 10.3390/cancers14030752 (PMC8833467; doi:10.3390/cancers14030752)
Supplement: Supplementary file 1 [file cancers-14-00752-s001.zip › cancers-1547617-supplementary.pdf]

Supplementary Materials

Table S1. Background data of patients in the treatment and no treatment groups.

	**Treatment group**	**No treatment group**	***p*-Value**
Number	96	31	
Age (years old)	69.6 (25.4–87.0)	73.0 (40.4–86.7)	0.189
Men	78 (81.3%)	24 (77.4%)	0.641
HBV	14 (14.6%)	5 (16.1%)	0.834
HCV	52 (54.2%)	17 (54.8%)	0.948
HBV+HCV	3 (3.1%)	0 (0.0%)	0.319
Alcoholic	13 (13.5%)	2 (6.5%)	0.288
NASH	8 (8.3%)	4 (12.9%)	0.449
Others	6 (6.3%)	3 (9.7%)	0.518
FIB-4 index	4.85 (0.48–27.8)	7.73 (1.44–39.5)	0.145
Child–Pugh score	5.5 (5 – 11)	8 (5 –13)	<0.001
ALBI score	−2.35 (−3.26 to −0.81)	−1.59 (−2.87 to −0.62)	<0.001
AFP (ng/mL)	107 (1–671940)	204 (1–245405)	0.818
DCP (mAU/mL)	506 (10–1084600)	1261 (22–562056)	0.040
Frequency of multiple intrahepatic HCC	70 (72.9%)	25 (80.6%)	0.389
Maximum size of intrahepatic HCC (mm)	38 (0–154)	58 (0–140)	0.003
Vascular invasion	55 (57.3%)	25 (80.6%)	0.019
Hepatic TNM	3 (0–4)	4 (0–4)	0.013
Performance status	0 (0–4)	0 (0–2)	0.665
Bone metastasis alone	32 (33.3%)	4 (12.9%)	0.028
Lung metastasis alone	13 (13.5%)	12 (38.7%)	0.002
Lymph node metastasis alone	16 (16.7%)	13 (41.9%)	0.004
another organ metastasis	5 (5.2%)	0 (0.0%)	0.195
Multiple metastases	30 (31.3%)	2 (6.5%)	0.006

AFP, α-fetoprotein; DCP, des-γ-carboxy prothrombin; HBV, hepatitis B virus; HCV, hepatitis C virus ; NASH, nonalcoholic steatohepatitis ; TNM, tumor-node-metastasis.

Table S2. Contents of TKI and conventional therapies.

	TKI group			Non-TKI group		*p*-Value
	**TKI alone**	**TKI plus conventional therapies**	Use of pembrolizumab or ramucirumab	Conventional therapies	TKI use before extrahepatic metastases	
n	29	36	2 (excluded)	25	4 (excluded)	
SOR	24	17	1	0	4	0.003 a)
LEN	4	12	0	0	0	0.069 a)
Multiple TKIs	1 SOR+LEN (1)	7 SOR+REG (1) SOR+LEN (5) SOR+REG+LEN (1)	1 SOR+REG (1)	0	0	0.051 a)
TACE/TAI		11		11		0.282 b)
RT		12		7		0.658 b)
TACE/TAI +RT		9		2		0.089 b)
other		4		5		0.336 b)

LEN, lenvatinib; REG, regorafenib; RT, radiation therapy; SOR, sorafenib; TACE, transcatheter arterial chemoembolization; TAI, transhepatic arterial infusion; other includes surgical resection and oral UFT (tegafur/uracil); TKI, Tyrosine kinase inhibitor. a) Comparison of TKI between TKI alone and TKI plus conventional therapies. b) Comparison of conventional therapies between TKI group and non-TKI group.

Table S3. Background data of patients in TKI and non-TKI groups.

	**TKI group**	**Non-TKI group**	***p*-Value**
Number	65	25	
Age (years old)	70.3 (25.4–85.9)	69.7 (49.6–87.0)	0.961
Men	54 (83.1%)	19 (76.0%)	0.442
FIB4-index	4.77 (0.48–27.8)	4.97 (1.74–25.7)	0.121
Child–Pugh score	5 (5 – 11)	6 (5 –10)	0.021
ALBI score	–2.41 (–3.25 to −0.90)	–2.03 (–3.26 to −0.81)	0.016
AFP (ng/mL)	63 (1–128650)	403 (2–671940)	0.140
DCP (mAU/mL)	395 (10–1084600)	1932 (16–562056)	0.077
Frequency of multiple intrahepatic HCC	45 (69.2%)	20 (80.0%)	0.307
Maximum size of intrahepatic HCC (mm)	35 (0–154)	41 (0–138)	0.114
Vascular invasion	30 (46.2%)	19 (76.0%)	0.011
Hepatic TNM	3 (0–4)	4 (0–4)	0.008
Performance status	0 (0–3)	0 (0–4)	0.250

AFP, α-fetoprotein; DCP, des-γ-carboxy prothrombin; TKI, Tyrosine kinase inhibitor; TNM, tumor-node-metastasis. .

Table S4. Background data of patients who started TKI after diagnosis of metastases.

	**TKI group**	**Non-TKI group**	***p*-Value**
Number	56	25	
Age (years old)	70.0 (45.9–85.9)	69.7 (49.6–87.0)	0.862
Men	46 (82.1%)	19 (76.0%)	0.521
FIB-4 index	4.19 (0.48–18.0)	4.97 (1.74–25.7)	0.076
Child–Pugh score	5 (5–11)	6 (5 –10)	0.035
ALBI score	−2.42 (−3.25 to −0.90)	−2.03 (−3.26 to −0.81)	0.016
AFP (ng/mL)	84 (1–128650)	403 (2–671940)	0.142
DCP (mAU/mL)	442 (10–1084600)	1932 (16–562056)	0.125
Frequency of multiple intrahepatic HCC	38 (67.9%)	20 (80.0%)	0.263
Maximum size of intrahepatic HCC (mm)	35 (0–154)	41 (0–138)	0.123
Vascular invasion	26 (46.4%)	19 (76.0%)	0.013
Hepatic TNM	3 (0–4)	4 (0–4)	0.009
Performance status	0 (0–3)	0 (0–4)	0.214

AFP, α-fetoprotein; DCP, des-γ-carboxy prothrombin; TKI, Tyrosine kinase inhibitor; TNM, tumor-node-metastasis.

Table S5. Background data of Child-Pugh A and B patients who started TKI after diagnosis of metastases.

	**Child-Pugh A**			**Child-Pugh B**		
	**TKI**	**Non-TKI**	**p-value**	TKI	Non- TKI	p-value
Number	46	15		9	9	
Age (years old)	69.6 (45.9–85.9)	75.5 (61.8–83.4)	0.115	73.9 (51.9–83.4)	63.7 (49.6–87.0)	0.301
Men	37 (80.4%)	11 (73.3%)	0.560	8 (88.9%)	7 (77.8%)	0.527
FIB-4 index	3.75 (0.48–18.0)	4.43 (1.74–14.3)	0.303	6.95 (3.75–17.2)	12.3 (3.58–25.7)	0.174
ALBI score	−2.49 (−3.25 to −1.83)	−2.38 (−3.26 to −1.65)	0.182	−1.92 (−2.21 to −1.19)	−1.77 (−2.44 to −0.90)	0.640
AFP (ng/mL)	57 (1–128650)	155 (4–369127)	0.248	504 (2–123578)	577 (2–671940)	0.659
DCP (mAU/mL)	352 (10–1084600)	166 (16–562056)	0.541	575 (23–82003)	5952 (24–45363)	0.825
Frequency of multiple intrahepatic HCC	30 (65.2%)	10 (66.7%)	0.918	7 (77.8%)	9 (100%)	0.134
Maximum size of intrahepatic HCC (mm)	26 (0–154)	41 (0–125)	0.322	52 (0–127)	53 (24–138)	0.750
Vascular invasion	19 (41.3%)	10 (66.7%)	0.088	6 (66.7%)	8 (88.9%)	0.257
Hepatic TNM	3 (0–4)	3 (0–4)	0.144	3 (0–4)	4 (3–4)	0.048
Performance status	0 (0–3)	0 (0–4)	0.736	0 (0–0)	0 (0–3)	0.066

AFP, α-fetoprotein; DCP, des-γ-carboxy prothrombin; TKI, Tyrosine kinase inhibitor; TNM, tumor-node-metastasis.

Table S6. Background data of patients who meet the REFLECT criteria (REFLECT IN) and do not meet the criteria (REFLECT OUT).

	**REFLECT IN**			REFLECT OUT		
	**TKI**	**TKI plus conventional**	**p-value**	TKI	TKI plus conventional	p-value
Number	14	21		15	15	
Age (years old)	72.2 (25.4–84.2)	69.1 (49.9–80.9)	0.893	71.1 (51.9–85.9)	67.5 (57.4–85.1)	0.788
Men	12 (85.7%)	16 (76.2%)	0.490	13 (86.7%)	13 (86.7%)	1.000
FIB4-index	3.81 (1.56–8.3)	3.60 (1.84–8.52)	0.731	8.63 (0.95–18.0)	6.29 (0.48–25.7)	0.443
Child–Pugh score	5 (5 – 6)	5 (5 – 6)	0.213	6 (5 –11)	5 (5 – 8)	0.145
ALBI score	−2.61 (−3.14 to −2.14)	−2.50 (−3.25 to −1.83)	0.730	−1.86 (−3.20 to −0.90)	−2.41 (−3.02 to −1.19)	0.086
AFP (ng/mL)	79 (1–127956)	14 (1–2132)	0.252	504 (2–128650)	70 (1–123578)	0.372
DCP (mAU/mL)	30 (10–5061)	216 (15–9129)	0.029	1832 (10–1084600)	464 (21–63989)	0.089
Frequency of multiple intrahepatic HCC	10 (71.4%)	12 (57.1%)	0.392	11 (73.3%)	12 (80.0%)	0.666
Maximum size of intrahepatic HCC (mm)	23 (0–57)	14 (0–78)	0.804	63 (25–154)	35 (0–120)	0.008
Vascular invasion	4 (28.6%)	4 (19.0%)	0.511	13 (86.7%)	9 (60.0%)	0.099
Hepatic TNM	3 (0–4)	2 (0–4)	0.593	4 (2–4)	3 (0–4)	0.139
Performance status	0 (0–1)	0 (0–1)	0.331	0 (0–3)	0 (0–2)	0.544
HCC occupation ≥50% of the liver	0 (0%)	0 (0%)	-	4 (26.7%)	1 (6.7%)	0.142
Bile duct invasion	0 (0%)	0 (0%)	-	3 (20.0%)	3 (20.0%)	1.000
Main portal vein invasion (Vp4)	0 (0%)	0 (0%)	-	1 (6.7%)	0 (0%)	0.309
Sorafenib	12	13	0.126	12	4	0.003
Lenvatinib	2	5	0.384	2	7	0.046
Multiple TKI	0	3	0.139	1	4	0.142

AFP, α-fetoprotein; DCP, des-γ-carboxy prothrombin; TKI, Tyrosine kinase inhibitor; TNM, tumor-node-metastasis.

Table S7. Background data of patients with lung or bone metastases.

	**Bone metastasis alone**	**Lung metastasis alone**	**p-value**
Number	32	13	
Age (years old)	69.7 (56.3–85.9)	67.1 (25.4–83.7)	0.582
Men	27 (84.4%)	11 (84.6%)	0.984
FIB-4 index	5.84 (0.95–27.8)	5.30 (1.56–25.6)	1.000
Child–Pugh score	6 (5–9)	6 (5–7)	0.925
ALBI score	−2.26 (−3.25 to −1.09)	−2.47 (−3.20 to −1.45)	0.903
AFP (ng/mL)	128 (1–36917)	247 (3–671940)	0.367
DCP (mAU/mL)	1545 (21–1084600)	4059 (10–43699)	0.802
Frequency of multiple intrahepatic HCC	24 (75.0%)	11 (84.6%)	0.482
Maximum size of intrahepatic HCC (mm)	43 (0–154)	41 (0–138)	0.573
Vascular invasion	20 (62.5%)	8 (61.5%)	0.952
Hepatic TNM	4 (0–4)	4 (0–4)	0.674

Performance status	0 (0–4)	0 (0–2)	0.428
TKI use	23 (71.9%)	10 (76.9%)	0.729

AFP, α-fetoprotein; DCP, des-γ-carboxy prothrombin; TKI, Tyrosine kinase inhibitor; TNM, tumor-node-metastasis.
